# Improving Phylogeny Reconstruction at the Strain Level Using Peptidome Datasets

**DOI:** 10.1371/journal.pcbi.1005271

**Published:** 2016-12-29

**Authors:** Aitor Blanco-Míguez, Jan P. Meier-Kolthoff, Alberto Gutiérrez-Jácome, Markus Göker, Florentino Fdez-Riverola, Borja Sánchez, Anália Lourenço

**Affiliations:** 1 ESEI–Department of Computer Science, University of Vigo, Edificio Politécnico, Campus Universitario As Lagoas s/n, Ourense, Spain; 2 Department of Microbiology and Biochemistry of Dairy Products, Instituto de Productos Lácteos de Asturias (IPLA), Consejo Superior de Investigaciones Científicas (CSIC), Villaviciosa, Asturias, Spain; 3 Leibniz Institute DSMZ–German Collection of Microorganisms and Cell Cultures GmbH, Inhoffenstraße 7B, Braunschweig, Germany; 4 CEB—Centre of Biological Engineering, University of Minho, Campus de Gualtar, Braga, Portugal; Centre for Research and Technology-Hellas, GREECE

## Abstract

Typical bacterial strain differentiation methods are often challenged by high genetic similarity between strains. To address this problem, we introduce a novel *in silico* peptide fingerprinting method based on conventional wet-lab protocols that enables the identification of potential strain-specific peptides. These can be further investigated using *in vitro* approaches, laying a foundation for the development of biomarker detection and application-specific methods. This novel method aims at reducing large amounts of comparative peptide data to binary matrices while maintaining a high phylogenetic resolution. The underlying case study concerns the *Bacillus cereus* group, namely the differentiation of *Bacillus thuringiensis*, *Bacillus anthracis* and *Bacillus cereus* strains. Results show that trees based on cytoplasmic and extracellular peptidomes are only marginally in conflict with those based on whole proteomes, as inferred by the established Genome-BLAST Distance Phylogeny (GBDP) method. Hence, these results indicate that the two approaches can most likely be used complementarily even in other organismal groups. The obtained results confirm previous reports about the misclassification of many strains within the *B*. *cereus* group. Moreover, our method was able to separate the *B*. *anthracis* strains with high resolution, similarly to the GBDP results as benchmarked via Bayesian inference and both Maximum Likelihood and Maximum Parsimony. In addition to the presented phylogenomic applications, whole-peptide fingerprinting might also become a valuable complementary technique to digital DNA-DNA hybridization, notably for bacterial classification at the species and subspecies level in the future.

## Author Summary

Molecular based differentiation of bacterial species is important in phylogenetic studies, diagnostics and epidemiological surveillance, particularly where unusual phenotype makes the classical phenotypic identification of bacteria difficult. Typical bacterial differentiation methods are often challenged by a high genetic similarity among strains. For decades, the technique of choice to classify and identify bacteria was DNA-DNA hybridization (DDH). The boosting of whole-genome sequencing technology facilitated the development of bioinformatics alternatives that could assist a much wider number of laboratories and are less biased to experimental errors. Currently, the Genome-to-Genome Distance Calculator web service, implementing the Genome-BLAST Distance Phylogeny (GBDP) method, provides the highest correlation to conventional DDH. Our methodology shows that whole peptide fingerprinting may complement the outputs of GBDP, i.e. experimental mass spectra may be used to cluster the bacteria, and more specifically it has been found useful for bacterial classification at the species and subspecies level. In addition, we present here how peptidome subsets obtained from in silico digestion of the peptidomes, is an efficient way to maintain the phylogenetic signal whilst reducing the total amount of data, making this methodology suitable for handling large data sets as in the case of epidemiologic studies.

This is a *PLOS Computational Biology* Methods paper.

## Introduction

The most common techniques for bacterial classification and identification are conventional DNA:DNA hybridization (DDH) [1], comparison of 16S or 23S rRNA gene sequences or 16S–23S rRNA spacer regions [2], multi-locus sequence typing (MLST) [3] and rep-PCR fingerprinting [4], among others [5]. For decades, the technique of choice to identify and classify species has been DDH with a similarity value of 70% DDH as the species delimitation threshold [6]. In microbial taxonomy, DDH is mandatory whenever the 16S rRNA gene sequence similarity between two strains is above 97% for confirming that these do not belong to the same species. This threshold has recently been increased by proposing values of between 98.2 and 99.0%, depending on the phylum [7]. Conventional DDH has limitations, for instance, that it is only available in a few specialized molecular laboratories world-wide and it is particularly biased to experimental errors [8]. Due to this and because of the availability of whole-genome sequencing, this facilitated the development of bioinformatics alternatives to conventional DDH [9].

Here, the Genome-to-Genome Distance Calculator web service (GGDC; freely available at http://ggdc.dsmz.de/) currently provides the highest in silico correlation to conventional DDH–without sharing the aforementioned drawbacks–which is a crucial requirement for any such in silico method to maintain consistency in prokaryotic species delineation [10]. The GGDC server incorporates the latest version [[10] of the Genome-BLAST Distance Phylogeny method (GBDP)—a highly optimized tool for the calculation of intergenomic distances—and estimates digital DNA-DNA hybridization values (dDDH values) from these distances under recommended settings [10]. Among other useful data, the dDDH values are reported along with confidence intervals, which are important for assessing the statistical uncertainty inherent to all model-based approaches [10]. In this way, GGDC can be reliably used for both species and subspecies delimitation [11].

The GBDP method incorporates several optimizations to avoid potentially biased results caused by elements such as paralogous genes or low-complexity regions. It is also robust against the use of incomplete genome sequences [10] and can be applied to both nucleotide and amino acid data. Finally, it includes a pseudo-bootstrapping procedure [10] for the calculation of replicate intergenomic distances, which can be further used in phylogenetic applications to assess branch support values as shown earlier [11–13].

Matrix Assisted Laser Desorption/Ionization Time Of Flight Mass Spectrometry (MALDI-TOF MS) has been applied as an alternative approach to identify and discriminate between species and strains [14–16]. This alternative is typically adopted when there is limited genetic variability within or across the species under study, and assumes the presence and detection of species/strain specific peptides through comparison of their mass-to-charge ratio. In this way this method supports species/strain differentiation. However, many of these differential peptides may not be detected due to their low abundance or other physicochemical properties, i.e., those methods are limited in such a way that it only explores a subset of the total peptidic variability.

To overcome this limitation, we have designed a novel *in silico* peptide fingerprinting methodology suitable for phylogeny inference. This methodology follows the same general principle of existing mass spectrometry approaches but it uses whole genome data and *in silico* protein digestion, i.e., it does not involve any conventional experimentation. Furthermore, the analysis stands on the shoulders of well-established software tools, namely PSortB [17], mzJava [18], SPECLUST [19] and MrBayes [20]. The aim is to be able to generate a valid and manageable list of peptides that are potentially specific to each strain. This list could then be further investigated using *in vitro* approaches, such as LC-MS/MS, towards the identification of biomarkers, strain specific peptides and the development of application-specific detection methods.

Our case study covers a subset of strains belonging to the *Bacillus cereus* group [21]. More precisely, the case study covers *B*. *thuringiensis*, *B*. *anthracis* and *B*. *cereus* (*senso stricto*) strains, which are known to share high genetic similarity [22]. Such strains are conventionally classified according to other features, such as their pathogenic potential or the presence of plasmids [23]. From a taxonomic point of view, separation of the three *Bacillus* species is still a subject of controversy among scientists. However, a recent large-scale whole-genome sequence-based study using GBDP elucidated the taxonomy within the *B*. *cereus* group and showed that *B*. *thuringiensis*, *B*. *anthracis* and *B*. *cereus* (*senso stricto*) species are indeed belonging to individual phylogenetic groups [12]. Other strains originally attributed to one of these three species, were either misclassified or belong to other novel species within the cluster. The results of the GBDP phylogenomic analysis serve as a good baseline, representative of what can currently be achieved with a state-of-the-art phylogenomic analysis as exemplified for the *B*. *cereus* group.

Currently, a method to infer bacterial taxonomy *in silico* through the use of peptidomes is missing. The development of such a method is appealing as it would complement GBDP analysis. Additionally, establishing the comparison and identification of unique peptides on an exemplary microbial data set would aid in the separation of closely related strains. Moreover, *in silico* peptidome fingerprinting is able to reduce whole proteome data into smaller binary matrices, which is of advantage when handling larger bacterial datasets. The amount of data may be decreased using different peptidome subsets without losing phylogenetic signal. Main results are discussed in this manuscript.

## Materials and Methods

**Fig 1:**
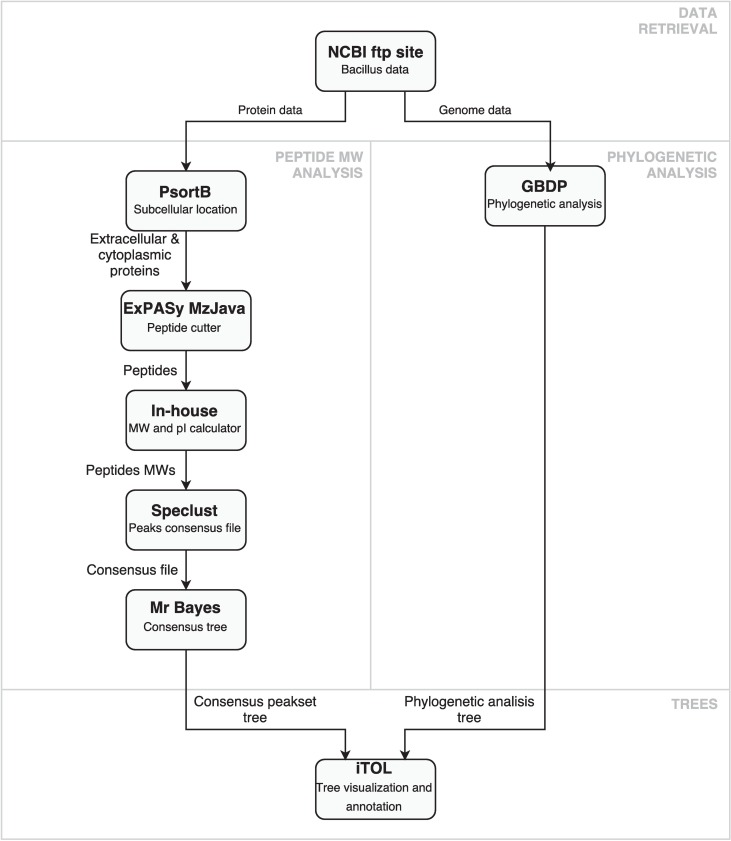
Peptidome-based genome comparison pipeline.

The following sections describe the methods and tools used in our peptidome-based strain-level genome comparison pipeline. These steps include the retrieval of proteins encoded in the comparison genomes, the prediction of the subcellular localization of the proteins, the digestion of proteins from different locations, the comparison of the peptides according to their mass and the subsequent computation of consensus peak sets. The software consisted of public, well-known tools and in-house customized scripts. Pipeline is depicted in Fig 1.

### Genome and protein data retrieval

All the sequence data used in this study were retrieved from the BioProject collection of the National Center for Biotechnology Information (NCBI), using their public FTP site (ftp://ftp.ncbi.nih.gov/genomes/bacteria/) [24]. Our study focused on the complete genomes of *Bacillus anthracis*, *Bacillus cereus* and *Bacillus thuringiensis* whose BioProject accession numbers are listed in Table 1. Genetic data was obtained from *.fna files, whereas proteomes for in silico digestion were obtained from *.faa archives. *Bacillus subtilis* subsp. *natto* BEST195 was selected as an outgroup. For efficiency and to increase the flexibility in the analyses, protein data were stored in an in-house database.

**Table 1:** The Bacillus strains used in this study. Genome and protein data were retrieved from the BioProject collection of the NCBI in July, 2015.

Bacillus strains	BioProject accession number
*B*. *anthracis str*. A0248	PRJNA59385
*B*. *anthracis str*. “Ames Antecesor”	PRJNA58083
*B*. *anthracis str*. Ames	PRJNA57909
*B*. *anthracis str*. CDC 684	PRJNA59303
*B*. *anthracis str*. H901	PRJNA162021
*B*. *anthracis str*. Sterne	PRJNA58091
*B*. *cereus* 03BB202	PRJNA59299
*B*. *cereus* AH187	PRJNA58757
*B*. *cereus* AH820	PRJNA58751
*B*. *cereus str*. ATCC 10987	PRJNA57673
*B*. *cereus str*. ATCC 14579	PRJNA57975
*B*. *cereus* B4264	PRJNA58757
*B*. *cereus biovar anthracis str*. CI 684	PRJNA50615
*B*. *cereus str*. E33L 10987	PRJNA58103
*B*. *cereus* F837/76 strain:F0837/76	PRJNA83611
*B*. *cereus* FRI-35	PRJNA173403
*B*. *cereus* G9842	PRJNA58759
*B*. *cereus* NC7401	PRJNA82815
*B*. *cereus str*.Q1	PRJNA58529
*B*. *subtilis subsp*. *natto* BEST195	PRJNA183001
*B*. *thuringiensis str*. Al Hakam	PRJNA58795
*B*. *thuringiensis* BMB171	PRJNA49135
*B*. *thuringiensis* Bt407	PRJNA177931
*B*. *thuringiensis* HD 771	PRJNA173374
*B*. *thuringiensis* HD 789	PRJNA173860
*B*. *thuringiensis* MC28	PRJNA176369
*B*. *thuringiensis serovar chinensis* CT-43	PRJNA158151
*B*. *thuringiensis serovar finitimus* YBT-020	PRJNA158875
*B*. *thuringiensis serovar thungiensis str*. IS5056	PRJNA190186
*B*. *thuringiensis serovar konkurian str*. 97–27	PRJNA58089
*B*. *thuringiensis serovar kurstaki str*. HD73	PRJNA189188
*B*. *thuringiensis* YBT-1518	PRJNA229419

### Protein subcellular localization prediction

Subcellular localization defines the putative localization of the protein in the cell. This information is relevant because, for instance, extracellular proteins are used by the bacterium to communicate with its environment and thereby could help in bacterial differentiation. The subcellular localizations of the proteins were predicted using the standalone version of the PSortB v3.0 tool, following the developer guidelines [17]. The subsets corresponding to chromosomal proteins and plasmids were stored in the in-house database.

### Peptidome generation

Bacterial proteomes were obtained for all the *Bacillus* strains used in this work. The open-source Java library mzJava from ExPASy (http://mzjava.expasy.org) supported protein digestion [18]. For the purposes of the present analysis, three proteases representing the major intestinal endoproteases were used: trypsin, chymotrypsin and pepsin (low specificity model, pH>2). Resulting peptides, denominated peptidomes, were also stored in the in-house database. Five different datasets were considered in our study: i) whole proteomes using GBDP for calculating intergenomic distances (GBDP), ii) peptides with a length > 28 amino acids obtained from cytoplasmic proteins (Cyto28-more), iii) peptides with a length comprised between 51 and 60 amino acids obtained from cytoplasmic proteins included in the pI range 4.5–5.5 (Cyto_PI_51–60), iv) peptides with a length higher than 60 amino acids obtained from cytoplasmic proteins included in the pI range 4.5–5.5 (Cyto_PI_60-more), and v) peptides obtained from extracellular proteins (Extracellular). For the four last subsets, three different methodologies were used to infer phylogenies, Bayesian (MB), Maximum Likelihood (ML) and Maximum Parsimony (MP).

### Consensus peak set

The consensus peak set among all the strains was obtained in two steps. First, the list of the total peptides for each strain was subdivided based on peptide length for indexing purposes. Then, the molecular weight and isoelectric point of the selected peptides were calculated using an in-house customised tool adapted from the SIB Bioinformatics Resource Portal (http://web.expasy.org/compute_pi/). In the case of peptides obtained from extracellular proteomes, all peptides were kept for analysis.

SPECLUST, a public web-based tool, was used to identify representative and reproducible peak masses that are present in a collection of spectral profiles [18]. This tool calculates the mass difference between two peaks taken from different peak lists and determines whether or not the two peaks are identical, taking into account some measurement uncertainty (σ). In the present study, the measurement uncertainty was set empirically to 3.0 Da. In addition, the pairwise cut-off was set to 0.6, i.e., a peak was considered shared between two spectra if it was matched in the alignment of the spectra with a peak match score greater than 0.6 (corresponding to a 0.5 Da mass difference). The consensus spectra matrix was translated to a binary matrix (0s and 1s, representing absence or presence of a given peptide mass respectively) in NEXUS file format [25].

### Tree reconstruction based on consensus peak set data

MrBayes, the model-based phylogenetic inference tool using Bayesian statistics, was utilised to generate a consensus tree [20]. The consensus binary file obtained from the previously generated SPECLUST consensus file was used as input. The phylogeny was inferred through the restriction data type implemented in MrBayes (with state 0 or 1 representing the absence or presence of a consensus peptide throughout the strain peptidomes). For the purpose of our study, we assumed that the frequencies of these two possible states had a Dirichlet (1.00, 1.00) prior parameter. Bayesian analysis was performed in two independent runs using four Markov chains and 1,000,000 generations. When necessary, the number of generations was incremented for chain convergence diagnosis. The potential scale-reduction factor, printed at the end of the analysis, was used as convergence diagnosis. A majority-rule consensus tree (50%) was obtained after discarding the initial 25% of the trees (burnin = 250), where the log-likelihood values of the analysis (log probability of the data given the parameter values) are frequently not yet stabilized. Using this command, MrBayes plots the number of generations (each corresponding to a phylogenetic tree) versus its log probability. Usually, the first sampled trees show trends towards increasing or decreasing log-likelihood values, which results in inadequate sampling from the posterior probability distribution

Maximum likelihood (ML) and maximum parsimony (MP) phylogenies were inferred using the DSMZ phylogenomics pipeline [11]. A multiple sequence alignment was created with MUSCLE [26], and ML and MP trees were inferred from it with RAxML [27] and TNT [28], respectively. For ML, rapid bootstrapping in conjunction with the autoMRE bootstopping criterion [29] and subsequent search for the best tree was used; for MP, 1000 bootstrapping replicates were used in conjunction with tree-bisection-and-reconnection branch swapping and ten random sequence addition replicates.

### GBDP-based phylogenomic analysis including (sub-)species clustering

A whole-genome phylogeny (based on the proteome data) was inferred using the latest version of the Genome-BLAST Distance Phylogeny (GBDP) method [11,30]. Here, pairwise proteome comparisons (including pseudo-bootstrap replicates) were done under the greedy-with-trimming algorithm and further recommended settings [13]. The tree was inferred using FastME v2.07 with TBR post-processing [31]. The species and subspecies clustering was conducted on the nucleotide data (i) with the help of the Genome-to-Genome Distance Calculator (GGDC), (ii) established (sub-)species distance cut-offs [11,12], and (iii) the OPTSIL clustering tool [32], in analogy to a recent study [12].

### Tree visualization and annotation

The Interactive Tree Of Life (iTOL) web-based tool was utilised to visualize the phylogenetic trees [33]. Using the tree files generated previously, the annotation was performed, highlighting the BCG (*Bacillus Cereus* Group) notation as reported before by Li et al. [12]. Posterior probabilities or branch support values were included when equal or above 60%.

### Inferred trees comparison

The inferred trees were compared amongst themselves and with the pseudo-bootstrapped whole-proteome GBDP phylogeny [13]. The topological comparison was based on pairwise weighted Robinson-Foulds distances, which were calculated using the RaxML tool [27,34]. Visualisation was supported by the packages ggplot [35] and ggdendro [36] for the statistical language R [37].

## Results and Discussion

**Fig 2:**
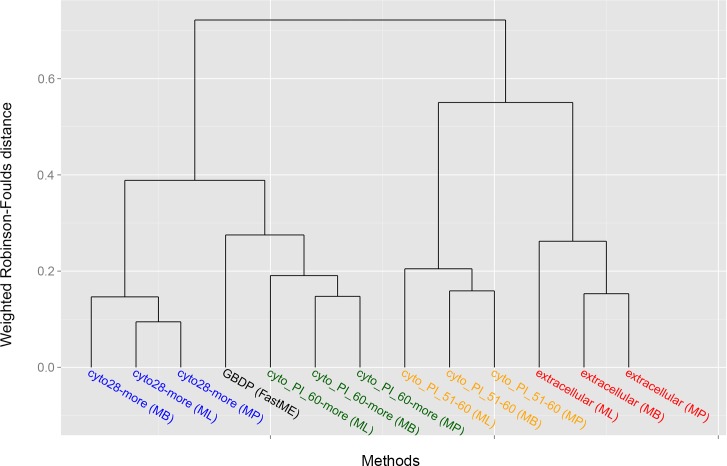
Dendrogram showing the hierarchical clustering of all pairwise weighted Robinson-Foulds distances using the Ward method [57]. Four main clusters are found, each one representing one of the four peptidome subsets. The proteome-based GBDP tree that was used as a baseline reference has the smallest distance to the [cytoPI60-more]-based trees and is thus closely positioned to that subgroup. The phylogenetic reconstruction methods FastME, ML, MP and MB are given in parentheses.

The results obtained with our *in silico* peptidome-based strain comparison pipeline are presented in Fig 2. These results reflect the analysis of the complete genomes of *B*. *anthracis*, *B*. *cereus* and *B*. *thuringiensis*, using *B*. *subtilis* subsp. natto BEST195 as outgroup (Table 1). We adopted the nomenclature proposed by Liu et al. regarding the taxonomy of *Bacillus cereus *[12]. Briefly, these authors used a GBDP analysis to separate 224 *Bacillus cereus* strains into 30 clusters. Eleven of these clusters represented already described species, whereas 19 clusters supported the proposal of putative novel species. These clusters where annotated as *Bacillus cereus* groups (BCG), and we have used such annotation in the present discussion.

Our analysis of the results is focused on intra-cluster strain homogeneity and the unexpected or incongruent grouping of certain strains. The rationale behind inter-cluster strain allocation agreement between the different approaches is that strain specialisation or evolution is likely to affect the peptide composition of the subcellular locations differently.

### GBDP phylogenomic analysis

**Fig 3:**
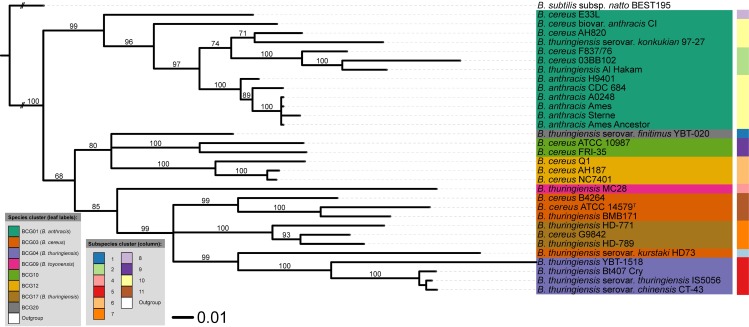
Whole proteome-based phylogenomic tree, including species and subspecies cluster information. This analysis was based on the GBDP algorithm and rooted with *Bacillus subtilis*. Numbers above branches are greedy-with-trimming pseudo-bootstrap support values from 100 replicates [13] and only support above 60% is shown. The leaf labels refer to the current NCBI nomenclature, whereas the BCG groups represent the recently revised names [12].

As illustrated in Fig 3, the GBDP proteome tree recovered all species with high support and showed insignificant subspecies conflicts. Most notably, this tree has an average branch support of 84.7% (Table 2) and confirms previous results of a nucleotide-based GBDP analysis [12]. Moreover, the OPTSIL clustering method [32] yielded eight species clusters as well as ten subspecies clusters (excluding the outgroup of *B*. *subtilis*). For instance, the cluster BCG01 contained some “*B*. *cereus”* and “*B*. *thuringiensis”* strains, which in fact belong to *B*. *anthracis* based on the dDDH estimates (see Supplementary S3 File). In turn, cluster BCG03 (*B*. *cereus*) included two “*B*. *thuringiensis”* strains: “*B*. *thuringiensis* BMB171” and “*B*. *thuringiensis* serovar *kurstaki* HD73". This is in accordance with a recent study on the taxonomic situation of the *B*. *cereus* group [12].

**Table 2:** Summary data on the different phylogenetic trees. Specifically, the average support obtained by the different methods and the size of the character matrices used.

Dataset	MrBayes	RaxML	TNT	GBDP	# characters
Cyto_PI_51–60	67.03	50.03	61.59	~	229
Extracellullar	80.03	65.93	72.93	~	530
Cyto_PI_60-more	84.13	76.07	79.48	~	1166
Cyto28-more	93.33	83.79	84.52	~	1696
GBDP	~	~	~	84.7	~

In summary, three major groups were identified: (i) BCG01 containing traditional and anomalously assigned strains of *B*. *anthracis*, (ii) a group encompassing the three related BCG03 (*B*. *cereus*), BCG04 (*B*. *thuringiensis*) and BCG17 and, (iii) a group formed by BCG10, BGC12 and BCG20 comprising three potential novel species [12]. Finally, “*B*. *thuringiensis* MC28” was classified into BCG09, which has been proposed as a novel species [12].

### Assessment of peptidome datasets

The phylogenies of the peptidome datasets resulting from all possible combinations of the three human proteases were evaluated based on MB, ML and MP criteria (see Supplementary S1 File). We also investigated proteins with different subcellular location as a possible way of reducing the amount of proteomic data input. In the case of extracellular proteins, all the resulting peptides were used in the analysis, but in the case of cytoplasmic peptidomes, the high number of peptides was further reduced by means of amino acid length and pI value filtering. Specifically, we considered three length bins, i.e. 28-more, 51–60 and 60-more amino acids, and those proteins with a pI between 4.5 and 5.5, which corresponds to the pI exhibited by most of the housekeeping and metabolic enzymes, as deduced from as deduced from 2 dimensional electrophoresis experiments [38]. In addition, genes coding for many of these proteins, such the β-subunit of RNA polymerase (*rpoB*), the β-subunit of ATP synthase F_0_F_1_ (*atpD*), or the chaperonin GroEL (*groEL*) are frequently used in multilocus sequence typing approaches [39]. Interestingly, this pI range do not correspond with the normal cytoplasmatic pH in mesophilic organisms such as Escherichia coli or Bacillus subtilis, which is slightly alkaline (7.0–7.8) over an external pH ranges of 5.0–9.0 [40–44] was determined by means of a flow cytometry with the fluorescent probe 5(and 6-)-carboxyfluorescein ester. As an example, we can say that the dataset including peptides with more than 60 amino acids comprised approximately 1,000 peptides per strain (Suppl. S2 File), which contrasts with the 320,000–411,000 peptides obtained after proteome digestion for the different strains concerned in this study, and results in an obvious reduction of data input.

**Fig 4:**
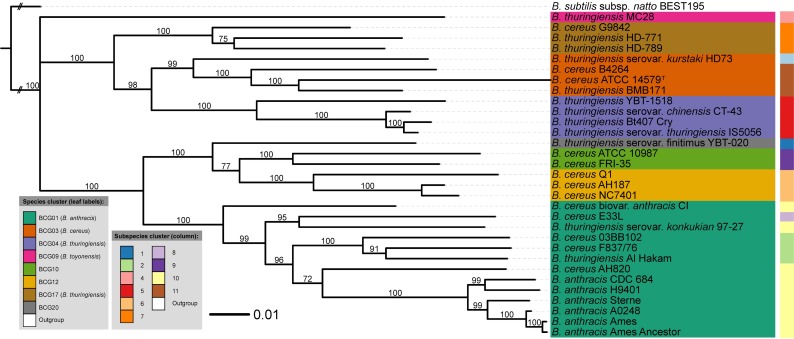
Bayesian tree based on the [Extracellular] dataset. Peptides were obtained from extracellular proteins. Bayesian analysis was performed in two independent runs using four Markov chains and 1,000,000 generations [58]. A majority-rule consensus tree (50%) was obtained after discarding the initial 25% of the trees and only support above 60% is shown. The leaf labels refer to the current NCBI nomenclature, whereas the BCG groups represent the recently revised names [12].

**Fig 5:**
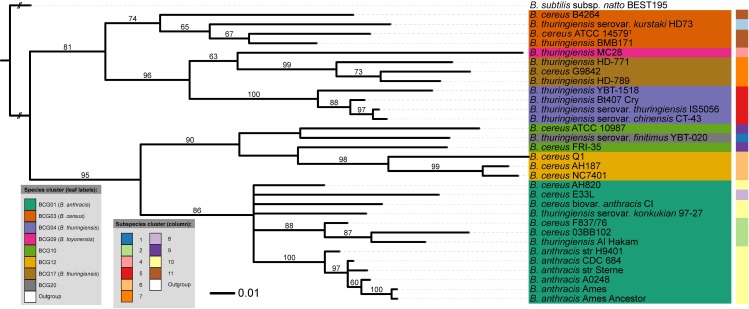
Bayesian tree based on [Cyto28-more] dataset. It contains peptides with a length higher or equal than 28 amino acids obtained from cytoplasmic. Bayesian analysis was performed in two independent runs using four Markov chains and 1,000,000 generations [58]. A majority-rule consensus tree (50%) was obtained after discarding the initial 25% of the trees and only support above 60% is shown. The leaf labels refer to the current NCBI nomenclature, whereas the BCG groups represent the recently revised names [12].

**Fig 6:**
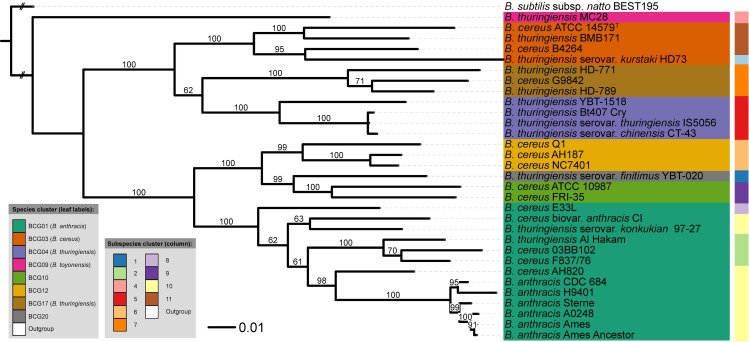
Bayesian tree based on [Cyto_PI_51–60] dataset. It contains peptides with 51–60 amino acids obtained from cytoplasmic proteins with an isoelectric point between 4.5 and 5.5. Bayesian analysis was performed in two independent runs using four Markov chains and 1,000,000 generations [58]. A majority-rule consensus tree (50%) was obtained after discarding the initial 25% of the trees and only support above 60% is shown. The leaf labels refer to the current NCBI nomenclature, whereas the BCG groups represents the recently revised names [12].

**Fig 7:**
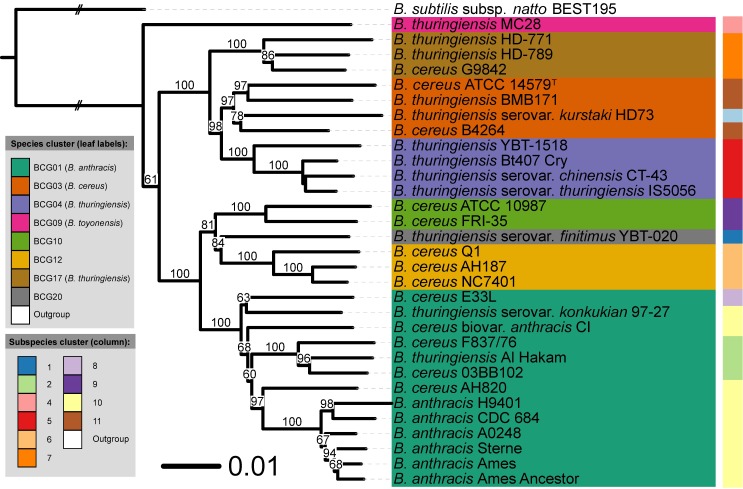
Bayesian tree based on [Cyto_PI_60-more] dataset. It contains peptides with a length higher than 60 amino acids obtained from cytoplasmic proteins with an isoelectric point between 4.5 and 5.5. Bayesian analysis was performed in two independent runs using four Markov chains and 1,000,000 generations [[58]. A majority-rule consensus tree (50%) was obtained after discarding the initial 25% of the trees and only support above 60% is shown. The leaf labels refer to the current NCBI nomenclature, whereas the BCG groups represent the recently revised names [12].

So, the hereafter presented results relate to the extracellular peptide dataset (Fig 4), the cytoplasmic dataset containing peptides with 28 or more amino acids (Fig 5), and the cytoplasmic datasets containing peptides with 51–60 amino acids or more than 60 amino acids and pI values within the range 4.5–5.5 (Figs 6 and 7, respectively). Interestingly, other filtering criteria, such as charge to mass amino acid ratio, may be implemented as mean as reducing the proteomic input.

### Phylogenomic inference based on peptidome datasets

The four peptide subsets were loaded in MrBayes and used to infer phylogenies. At the end of the Bayesian analysis, the average standard deviation of split frequencies after 1e06 generations suggested a good convergence of the analyses, as in all cases it was lower than 0.01 (Cyto28-more: 0.004; Cyto_PI_51–60: 0.007; Cyto_PI_60-more: 0.004; Extracellular: 0.006). Convergence of the analyses was confirmed by calculating the potential scale reduction factor (PSRF) of the total tree length (TL) and the stationary phase frequencies (pi) of the two possible states of our binary model (0 or 1). In all cases the PSRF values converged to 1.000–1.001 at the end of the analysis, indicating a good phylogenetic tree sampling from the posterior distribution.

A summary of the results of the phylogenetic inference is found in Table 2. The ML analyses yielded and subsequently used “Uncorrected+GAMMA” as best model during the inference. Since the ML, MP and MB trees were very similar within each peptidome dataset in terms of weighted topological distance (see below), only the MB-based trees are shown while discussing the different datasets. The remaining ML and MP trees are shown in Suppl. S1 File.

### Assessing conflicts among the inferred phylogenies

Pairwise weighted Robinson-Foulds distances supported the assessment of topological differences among the five trees at the light of the four methods of analysis (Fig 2). More specifically, the differences observed between the trees inferred from whole proteomes (GBDP analysis) and Cyto28-more, cyto_PI_60-more cyto_PI_51–60 and Extracellular subsets (applying the MB, ML and MP criteria). Significance of conflict between two trees was assumed when a bipartition implied by one tree was found incompatible with a bipartition implied by the other tree, with both receiving ≥95% support. Similarly, disagreement with the monophyly of a species or subspecies was only considered if the conflicting branches had ≥95% support.

As an initial observation we can say that the MB [Cyto_PI_60-more] and [Cyto28-more] trees showed no significant conflict with the GBDP tree. However, there are some interesting discrepancies between several trees. For example, in contrast to ML and MP trees, the MB [Cyto28-more] tree (Fig 5) showed significant conflict in terms of subspecies assignments within BCG01 (*B*. *anthracis*) cluster. Another example is the conflict between the MB [Extracellular] tree and some of the MB cytoplasmic trees regarding the placement of “*B*. *thuringiensis* serovar *kurstaki* HD73”. Specifically, in the [Extracellular] tree (Fig 4) the “*B*. *thuringiensis* serovar *kurstaki* HD73” is placed next to the BCG04 (*B*. *thuringiensis*) cluster with high support while in the MB [Cyto_PI_60-more] (Fig 6) and [Cyto28-more] (Fig 5) trees it is part of the BCG03 (*B*. *cereus*) group. Likewise, the MB [Cyto_PI_51–60] tree (Fig 6) significantly deviated from the GBDP proteome tree by placing *B*. *anthracis* H9401 as sister group of all other highly virulent *B*. *anthracis* strains instead of as sister group of *B*. *anthracis* CDC 684; and, by forming a well-supported group (96%) comprising “*B*. *thuringiensis* MC28”, the cluster BCG04 (*B*. *thuringiensis*) and the cluster BCG17 (*B*. *thuringiensis*). Noteworthy, these arrangements received no support in the ML and MP analyses of the [Cyto_PI_51–60] dataset. See Supplementary S1 File for details.

### Possible biological reasons behind conflicting phylogenetic grouping

The comparison of the peptidome-based phylogenetic trees allowed us to gain a better understanding about the information provided by the different sets of peptides. The four peptide subsets produced similar results regarding the identification of quite unrelated strains (e.g., *B*. *subtilis* subsp. *natto* BEST195), and established a species grouping as close as the one suggested by Liu et al. using 224 genomes of strains belonging to the *B*. *cereus* group [12]. Classically, *B*. *thuringiensis* strains have been considered an insect pathogen, affecting mainly members of the orders Lepidoptera, Diptera and Coleoptera [23]. Spores from these strains include large crystal protein inclusions, which are cleaved by the insect mid-gut proteases producing the active toxin forms. The action of this toxin leads to the complete destruction of the intestinal epithelium.

In turn, the BCG03 cluster corresponds to *B*. *cereus*, which is an opportunistic human pathogen and food-borne bacterium that causes two forms of poisoning, one characterised by diarrhea and abdominal pain, and the other involving nausea and vomiting [45,46]. Some *“B*. *thuringiensis”* strains also clustered in BCG03, because they share certain genetic similarity with *B*. *cereus* ATCC 14579^T^, namely genetic regions such as a putative polysaccharide capsule cluster [47].

*B*. *anthracis* (BCG01 cluster) is the etiological agent of anthrax, a fatal disease for herbivores and mammals that is best known for its use as biological weapon [48]. Strains from this species can be classified according to different phenotypical tests. For instance, these strains are non-motile, penicillin-sensitive, and produce an extracellular capsule of poly-γ-D-glutamic acid [49]. Toxins responsible for anthrax symptoms and other virulence factors necessary for complete virulence are codified into two large plasmids, denominated pXO1 and pXO2 [50]. Two strains of “*B*. *thuringiensis*” also clustered within the BCG01: “*B*. *thuringiensis* Al Hakam”, and “*B*. *thuringiensis* serovar *konkurian”*. Indeed both strains have been shown to be more related to the *B*. *anthracis* cluster. The genome of these strains contain no homologues of the known *B*. *thuringiensis* insecticidal genes *cry*, *cyt*, or *vip* and, even if these ever existed, the plasmid(s) encoding for these genes may have been lost during *in vitro* culture [50,51]. Therefore, classification of these two strains as *B*. *thuringiensis* strains may not be correct, as previously reported in [12].

Other cluster identified in our analysis was BCG17, a putative novel species. This contained “*B*. *cereus* G9842” together with other two “*B*. *thuringiensis*” strains. The G9842 strain was isolated from stool samples of an emetic outbreak that involved three individuals in Nebraska (1996) and the genome was sequenced by the J. Craig Venter Institute (http://www.ncbi.nlm.nih.gov/bioproject/17733). The isolate was characterised by MLST typing using the MLSTDB scheme as sequence type 56 (http://pubmlst.org/bcereus/). Interestingly, the sequence type 56 was quite unrelated to the major clade of pathogenic *B*. *cereus* isolates and was suggested as representative for a novel pathogenicity group within the *B*. *cereus* group [52]. Peptidome fingerprinting confirms the new affiliation *to B*. *thuringiensis*. The peptidome of strain G9842, shared a high homology with the other “*B*. *thuringiensis* strains”, so it is plausible that these two isolates lost the plasmids containing the insecticide genes and acquired certain virulence factors, which allow them to act as pathogens in the human host.

Finally, phylogenetic techniques consistently grouped “*B*. *thuringiensis* serovar *finitimus”* individually, and it has been proposed as representative for the novel species BCG20 [12]. This strain contains several *cry* genes encoding for crystal proteins and located in two plasmids [53]. The chromosome of this strain has been shown to be closer phylogenetically to *B*. *anthracis* Ames than to *B*. *cereus* ATCC 14579^T^ [12,54]. Given the close distance of “*B*. *thuringiensis* serovar *finitimus”* to the other BCG groups containing “*B*. *cereus”* strains, such as BCG10 and BCG12, we speculate that this strain may be a *B*. *cereus* strain that acquired the plasmids from a *B*. *thuringiensis* donor.

### Peptidome analysis benchmarking

Another important aspect of the evaluation of our peptidome similarity method is the computational complexity induced by each processing step and the resulting processing time eventually, although available computational power will be of course decisive. The running time of future *in silico* experiments can thus be extrapolated, especially that of significantly larger datasets. We computed the processing time of each of the main steps for the whole *Bacillus* dataset (i.e. 32 genomes) and for four subsets, representing a large dataset (i.e. 24 genomes), a medium-large dataset (i.e. 16 genomes), a medium-small dataset (i.e. 8 genomes) and a small dataset (i.e. 4 genomes, which is the smallest possible dataset that one can use for phylogenetic inference). In particular, we randomly sampled without replacement four sets of 24, 16, 8 and 4 genomes, and calculated the average running time. Here, we present the average times, but details on the different runs can be found in S4 File.

Table 3 summarises the running times taken by the steps of protein localization, which is performed by PsortB, and protein digestion, which is performed by ExPASy MzJava. Protein localization is the most time consuming task and, in particular, the processing of larger datasets may take several days. Although this may be considered somewhat time consuming, this step enables further filtering of the peptide dataset that, in turn, may reduce considerably the data matrices to be computed and speed up the subsequent steps of analysis.

**Table 3:** Running times for classification and protein digestion tasks in Bacillus cereus benchmarking. Running time is displayed using hours:minutes:seconds format.

Dataset	PSortB average running time	MZJava average running time	TOTAL average running time
All genomes*	56:42:07	0:49:45	57:31:52
24 genomes	42:37:48 +- 0:35:24	0:37:26 +- 0:00:49	43:15:14 +- 0:35:19
16 genomes	28:19:39 +- 0:15:17	0:24:37 +- 0:00:57	28:44:16 +- 0:15:57
8 genomes	13:51:25 +- 0:24:28	0:12:42 +- 0:01:10	14:04:07 +- 0:25:12
4 genomes	6:53:02 +- 0:09:10	0:06:16 +- 0:00:35	7:03:08 +- 0:13:43

*No replicates were performed.

The running times of steps leading to the generation of the NEXUS files are negligible compared to those of previous steps (Table 4). For most of the sample sets both steps took less than 15 minutes to execute. A large running time (> 2 hours) was observed for the SPECLUST run over the whole dataset of cytoplasmic peptides with 28 or more amino acids, which comprises a total of 121,632 peptides.

**Table 4:** Summary data on the NEXUS generation process for Bacillus cereus benchmarking. Running time is displayed using hours:minutes:seconds format.

Dataset	Settings			Speclust average running time	MrBayes average running time	TOTAL average running time
	Subcellular location	Isoelectric Point	Peptide Size			
All genomes	Extracellular	All	All	0:22:11	0:13:51	0:36:02
	Cytoplasmatic	4.5–5.5	50–60	0:05:47	0:05:42	0:11:29
	Cytoplasmatic	All	50–60	0:18:24	0:04:27	0:22:51
	Cytoplasmatic	4.5–5.5	60-more	0:05:13	0:17:41	0:22:54
	Cytoplasmatic	All	60-more	0:16:38	0:23:33	0:40:11
	Cytoplasmatic	4.5–5.5	28-more	0:20:41	0:16:33	0:37:14
	Cytoplasmatic	All	28-more	2:08:16	0:22:08	2:30:24
24 genomes	Extracellular	All	All	0:10:01	0:09:50	0:19:51
	Cytoplasmatic	4.5–5.5	50–60	0:03:10	0:04:29	0:07:40
	Cytoplasmatic	All	50–60	0:11:13	0:03:36	0:14:49
	Cytoplasmatic	4.5–5.5	60-more	0:04:07	0:13:14	0:17:21
	Cytoplasmatic	All	60-more	0:13:20	0:17:32	0:30:52
	Cytoplasmatic	4.5–5.5	28-more	0:11:13	0:12:45	0:23:58
	Cytoplasmatic	All	28-more	1:13:45	0:16:32	1:30:17
16 genomes	Extracellular	All	All	0:05:05	0:06:23	0:11:28
	Cytoplasmatic	4.5–5.5	50–60	0:02:23	0:03:48	0:06:11
	Cytoplasmatic	All	50–60	0:07:55	0:02:02	0:09:57
	Cytoplasmatic	4.5–5.5	60-more	0:02:09	0:08:29	0:10:38
	Cytoplasmatic	All	60-more	0:05:13	0:10:56	0:16:09
	Cytoplasmatic	4.5–5.5	28-more	0:04:38	0:07:57	0:12:35
	Cytoplasmatic	All	28-more	0:31:54	0:11:13	0:43:07
8 genomes	Extracellular	All	All	0:01:22	0:01:57	0:03:19
	Cytoplasmatic	4.5–5.5	50–60	0:00:54	0:01:35	0:02:29
	Cytoplasmatic	All	50–60	0:03:17	0:01:21	0:04:38
	Cytoplasmatic	4.5–5.5	60-more	0:01:15	0:02:17	0:03:32
	Cytoplasmatic	All	60-more	0:04:04	0:02:26	0:06:31
	Cytoplasmatic	4.5–5.5	28-more	0:01:47	0:01:52	0:03:39
	Cytoplasmatic	All	28-more	0:12:05	0:02:04	0:14:09
4 genomes	Extracellular	All	All	0:00:39	0:00:47	0:01:25
	Cytoplasmatic	4.5–5.5	50–60	0:00:59	0:00:37	0:01:36
	Cytoplasmatic	All	50–60	0:01:33	0:00:37	0:02:10
	Cytoplasmatic	4.5–5.5	60-more	0:00:27	0:00:35	0:01:01
	Cytoplasmatic	All	60-more	0:01:26	0:00:42	0:02:08
	Cytoplasmatic	4.5–5.5	28-more	0:00:35	0:00:49	0:01:24
	Cytoplasmatic	All	28-more	0:02:46	0:01:01	0:03:47

### Concluding remarks

One of the potential applications of our pipeline is to accept, as input, experimental peptide mass profiles. If traced back, our application allows detection of differential peptide profiles, providing a robust tool to discriminate not only strain-specific peptides, but true intraspecies differences among a set of biological replicates or even microorganism-phenotype variations such as those occurring between biofilm and planktonic populations. In this regard, the negative effect of certain peptide families on bacteria through different mechanisms is well known [55,56]. In this regard, our pipeline will just provide a candidate peptide list, but experimental approaches such as MS/MS experiments will never detect peptides that are inhibiting own bacterial growth. Rather, such experimental approaches will validate the presence of those certain strain-specific peptides, either free or most probably encoded in a “carrier protein”.

Generation of a potential strain-specific peptide list together with its experimental identification, may facilitate development of different approaches focused on the identification of given strain, such as a dairy starter or a probiotic that has to be traced through the human gut during clinical intervention studies. This can be accomplished, for instance, with the use of high-resolution mass spectrometers or antibody-based protocols targeting these specific peptides.

Whereas our bioinformatic approach will reliably produce the same results, conventional methods might yield different results even if applied on the same organisms, due for instance to phenotype-variations or the use of transient input data. In addition, the big advantage of the in silico method is accuracy, reproducibility and speed, whereas the disadvantage is that it might not get the experimental peptidome as we simply consider all proteins encoded in a genome and not only those that are actively produced by the organism while being measured.

Overall, results show that our phylogenetic method based on peptidome similarity, as opposed to genome-sequence homology, is complementary to the proteome-based GBDP analysis. Most notably, our peptidome-based phylogeny analysis supported already reported taxonomic discrepancies within the *B*. *cereus* group. Our peptidome-based method has the advantage of reducing larger amounts of proteomic data to small matrices (by a factor of 320) without losing too much phylogenetic signal. Our pipeline can be also applied to other peptide datasets originated from viruses, eukaryotic species or even metaproteomes with the inclusion of few modifications regarding the prediction of the protein subcellular location. This could be of interest for developing more efficient applications aimed at managing very large bacterial datasets, such as those generated in epidemiologic studies.

## Supporting Information

S1 File Set of additional phylogenetic trees (ML and MP) based on the digestion of one or two of the endoproteases considered in this study. (PDF)

S2 File Peptide distribution by subcellular localization and length. Ranges with few instances (e.g., loose ranges of extracellular peptides) and with high abundance (e.g., cytoplasmic proteins consisting of 11 to 20 amino acids) were not helpful for comparison. The analysis focused on extracellular peptides and cytoplasmic peptides consisting of 51 to 60 amino acids and above.
(PDF)

S3 File All pairwise digital DDH values regarding the 32 genomes dataset as calculated under the recommended settings of the GGDC 2.1. (ODS)

S4 File Peptidome analysis benchmarking. (XLSX)
