# Supplementary material for: Improving Phylogeny Reconstruction at the Strain Level Using Peptidome Datasets
Source: PLoS Comput Biol. 2016 Dec 29;12(12):e1005271. doi: 10.1371/journal.pcbi.1005271 (PMC5198984; doi:10.1371/journal.pcbi.1005271)
Supplement: S1 File — (PDF) [file pcbi.1005271.s001.pdf]

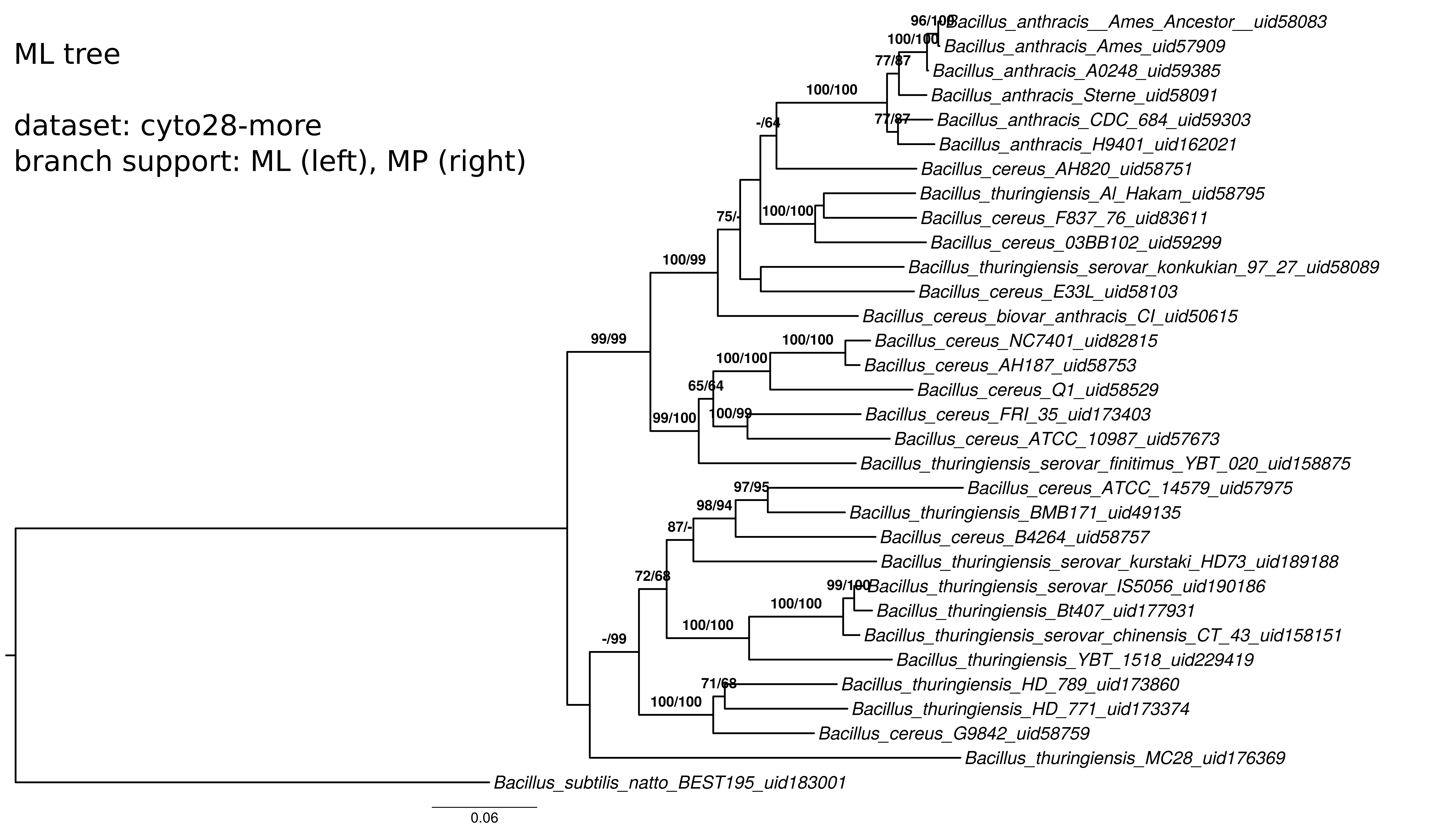
ML tree
dataset: cyto28-more
branch support: ML (left), MP (right)
96/100
Bacillus_anthracis__Ames_Ancestor__uid58083
100/100
Bacillus_anthracis_Ames_uid57909
77/87
Bacillus_anthracis_A0248_uid59385
100/100
Bacillus_anthracis_Sterne_uid58091
77/87
Bacillus_anthracis_CDC_684_uid59303
-/64
Bacillus_anthracis_H9401_uid162021
Bacillus_cereus_AH820_uid58751
Bacillus_thuringiensis_Al_Hakam_uid58795
100/100
Bacillus_cereus_F837_76_uid83611
75/-
Bacillus_cereus_03BB102_uid59299
100/99
Bacillus_thuringiensis_serovar_konkukian_97_27_uid58089
Bacillus_cereus_E33L_uid58103
Bacillus_cereus_biovar_anthracis_CI_uid50615
99/99
100/100
Bacillus_cereus_NC7401_uid82815
100/100
Bacillus_cereus_AH187_uid58753
65/64
Bacillus_cereus_Q1_uid58529
100/99
Bacillus_cereus_FRI_35_uid173403
99/100
Bacillus_cereus_ATCC_10987_uid57673
Bacillus_thuringiensis_serovar_finitimus_YBT_020_uid158875
97/95
Bacillus_cereus_ATCC_14579_uid57975
98/94
Bacillus_thuringiensis_BMB171_uid49135
87/-
Bacillus_cereus_B4264_uid58757
Bacillus_thuringiensis_serovar_kurstaki_HD73_uid189188
72/68
99/100
Bacillus_thuringiensis_serovar_IS5056_uid190186
100/100
Bacillus_thuringiensis_Bt407_uid177931
100/100
Bacillus_thuringiensis_serovar_chinensis_CT_43_uid158151
-/99
Bacillus_thuringiensis_YBT_1518_uid229419
71/68
Bacillus_thuringiensis_HD_789_uid173860
100/100
Bacillus_thuringiensis_HD_771_uid173374
Bacillus_cereus_G9842_uid58759
Bacillus_thuringiensis_MC28_uid176369
Bacillus_subtilis_natto_BEST195_uid183001
0.06

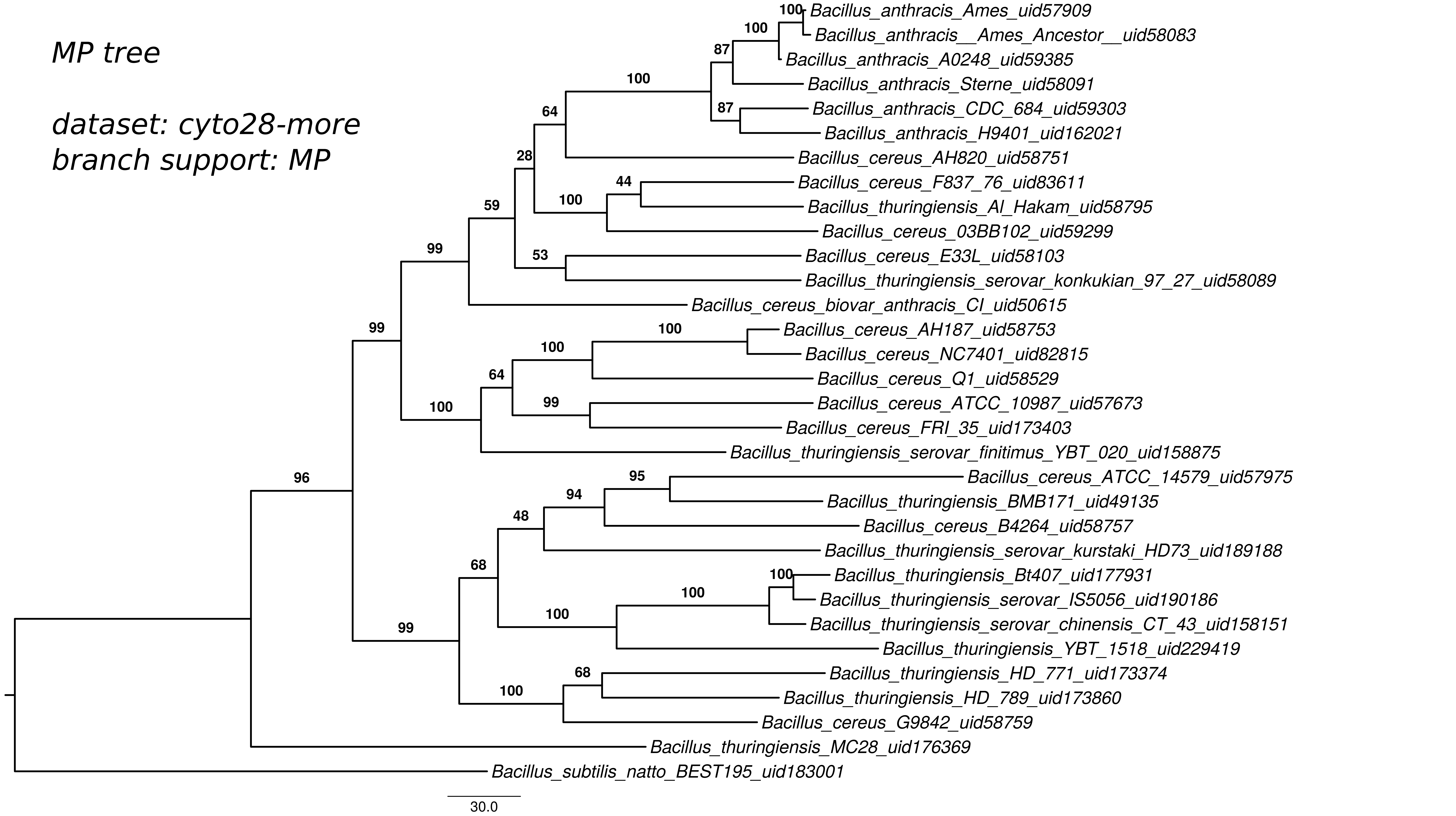
MP tree
dataset: cyto28-more
branch support: MP
Bacillus_anthracis_Ames_uid57909
Bacillus_anthracis__Ames_Ancestor__uid58083
Bacillus_anthracis_A0248_uid59385
Bacillus_anthracis_Sterne_uid58091
Bacillus_anthracis_CDC_684_uid59303
Bacillus_anthracis_H9401_uid162021
Bacillus_cereus_AH820_uid58751
Bacillus_cereus_F837_76_uid83611
Bacillus_thuringiensis_Al_Hakam_uid58795
Bacillus_cereus_03BB102_uid59299
Bacillus_cereus_E33L_uid58103
Bacillus_thuringiensis_serovar_konkukian_97_27_uid58089
Bacillus_cereus_biovar_anthracis_CI_uid50615
Bacillus_cereus_AH187_uid58753
Bacillus_cereus_NC7401_uid82815
Bacillus_cereus_Q1_uid58529
Bacillus_cereus_ATCC_10987_uid57673
Bacillus_cereus_FRI_35_uid173403
Bacillus_thuringiensis_serovar_finitimus_YBT_020_uid158875
Bacillus_cereus_ATCC_14579_uid57975
Bacillus_thuringiensis_BMB171_uid49135
Bacillus_cereus_B4264_uid58757
Bacillus_thuringiensis_serovar_kurstaki_HD73_uid189188
Bacillus_thuringiensis_Bt407_uid177931
Bacillus_thuringiensis_serovar_IS5056_uid190186
Bacillus_thuringiensis_serovar_chinensis_CT_43_uid158151
Bacillus_thuringiensis_YBT_1518_uid229419
Bacillus_thuringiensis_HD_771_uid173374
Bacillus_thuringiensis_HD_789_uid173860
Bacillus_cereus_G9842_uid58759
Bacillus_thuringiensis_MC28_uid176369
Bacillus_subtilis_natto_BEST195_uid183001
30.0

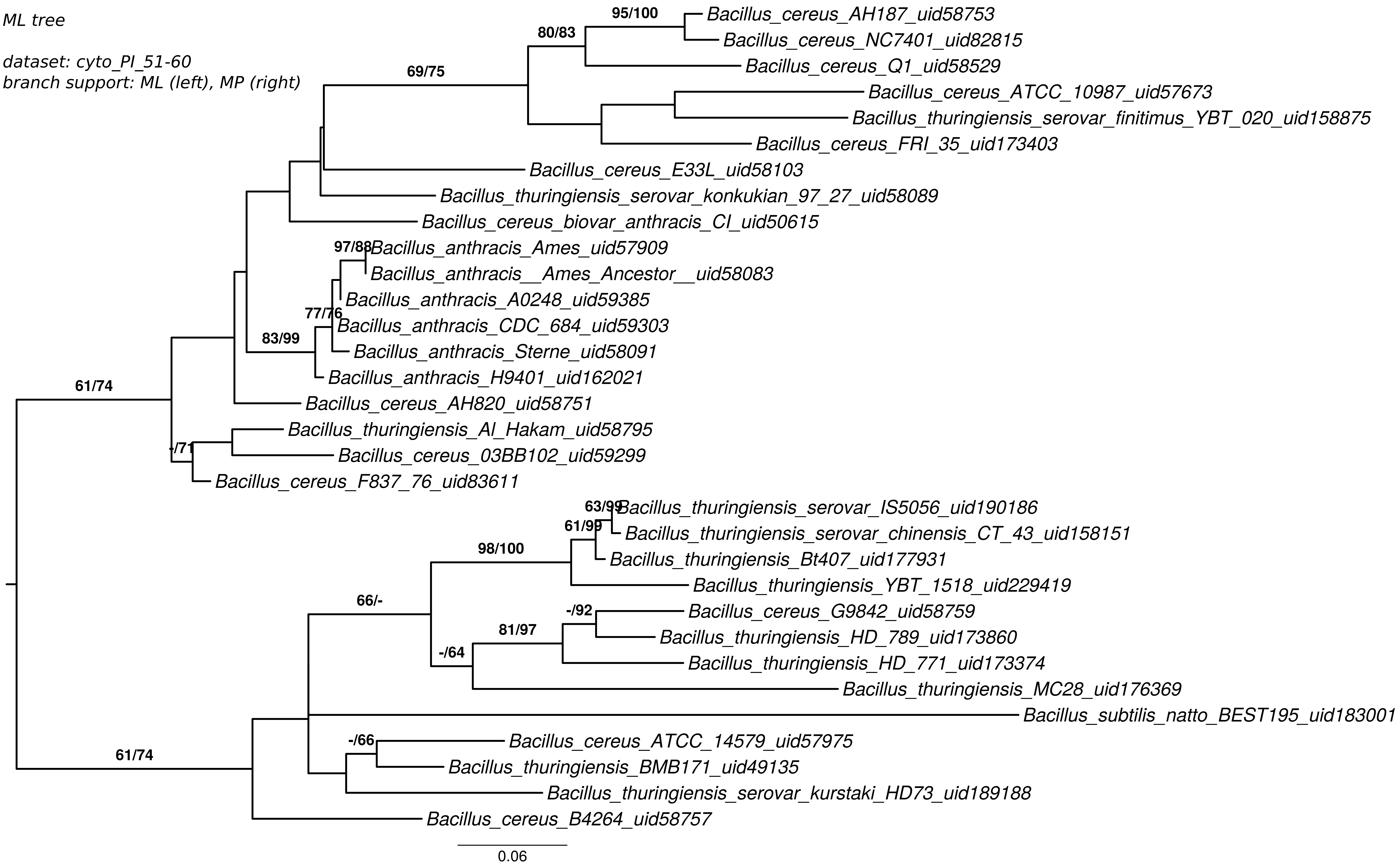
ML tree
dataset: cyto_PI_51-60
branch support: ML (left), MP (right)
95/100
80/83
69/75
Bacillus_cereus_AH187_uid58753
Bacillus_cereus_NC7401_uid82815
Bacillus_cereus_Q1_uid58529
Bacillus_cereus_ATCC_10987_uid57673
Bacillus_thuringiensis_serovar_finitimus_YBT_020_uid158875
Bacillus_cereus_FRI_35_uid173403
Bacillus_cereus_E33L_uid58103
Bacillus_thuringiensis_serovar_konkukian_97_27_uid58089
Bacillus_cereus_biovar_anthracis_CI_uid50615
97/88
Bacillus_anthracis_Ames_uid57909
Bacillus_anthracis__Ames_Ancestor__uid58083
Bacillus_anthracis_A0248_uid59385
77/76
Bacillus_anthracis_CDC_684_uid59303
83/99
Bacillus_anthracis_Sterne_uid58091
Bacillus_anthracis_H9401_uid162021
61/74
Bacillus_cereus_AH820_uid58751
Bacillus_thuringiensis_Al_Hakam_uid58795
-/71
Bacillus_cereus_03BB102_uid59299
Bacillus_cereus_F837_76_uid83611
63/99
Bacillus_thuringiensis_serovar_IS5056_uid190186
61/99
Bacillus_thuringiensis_serovar_chinensis_CT_43_uid158151
98/100
Bacillus_thuringiensis_Bt407_uid177931
Bacillus_thuringiensis_YBT_1518_uid229419
66/-
-/92
Bacillus_cereus_G9842_uid58759
81/97
Bacillus_thuringiensis_HD_789_uid173860
-/64
Bacillus_thuringiensis_HD_771_uid173374
Bacillus_thuringiensis_MC28_uid176369
Bacillus_subtilis_natto_BEST195_uid183001
-/66
Bacillus_cereus_ATCC_14579_uid57975
Bacillus_thuringiensis_BMB171_uid49135
61/74
Bacillus_thuringiensis_serovar_kurstaki_HD73_uid189188
Bacillus_cereus_B4264_uid58757
0.06

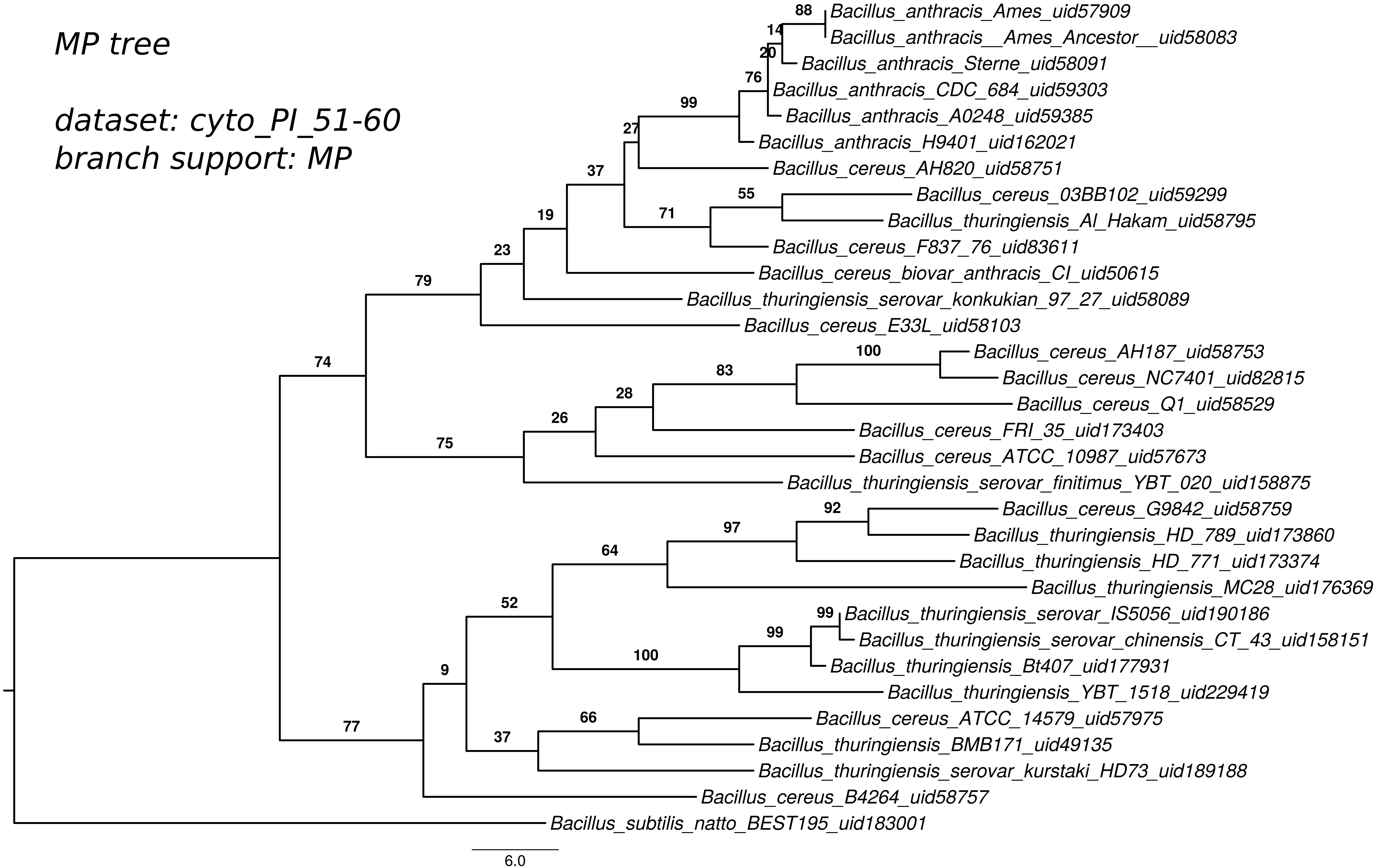
MP tree
dataset: cyto_PI_51-60
branch support: MP
Bacillus_anthracis_Ames_uid57909
Bacillus_anthracis__Ames_Ancestor__uid58083
Bacillus_anthracis_Sterne_uid58091
Bacillus_anthracis_CDC_684_uid59303
Bacillus_anthracis_A0248_uid59385
Bacillus_anthracis_H9401_uid162021
Bacillus_cereus_AH820_uid58751
Bacillus_cereus_03BB102_uid59299
Bacillus_thuringiensis_Al_Hakam_uid58795
Bacillus_cereus_F837_76_uid83611
Bacillus_cereus_biovar_anthracis_CI_uid50615
Bacillus_thuringiensis_serovar_konkukian_97_27_uid58089
Bacillus_cereus_E33L_uid58103
Bacillus_cereus_AH187_uid58753
Bacillus_cereus_NC7401_uid82815
Bacillus_cereus_Q1_uid58529
Bacillus_cereus_FRI_35_uid173403
Bacillus_cereus_ATCC_10987_uid57673
Bacillus_thuringiensis_serovar_finitimus_YBT_020_uid158875
Bacillus_cereus_G9842_uid58759
Bacillus_thuringiensis_HD_789_uid173860
Bacillus_thuringiensis_HD_771_uid173374
Bacillus_thuringiensis_MC28_uid176369
Bacillus_thuringiensis_serovar_IS5056_uid190186
Bacillus_thuringiensis_serovar_chinensis_CT_43_uid158151
Bacillus_thuringiensis_Bt407_uid177931
Bacillus_thuringiensis_YBT_1518_uid229419
Bacillus_cereus_ATCC_14579_uid57975
Bacillus_thuringiensis_BMB171_uid49135
Bacillus_thuringiensis_serovar_kurstaki_HD73_uid189188
Bacillus_cereus_B4264_uid58757
Bacillus_subtilis_natto_BEST195_uid183001
88
14
20
76
99
27
37
55
71
19
23
79
74
100
83
28
26
75
92
97
64
52
99
99
100
9
66
37
77
6.0

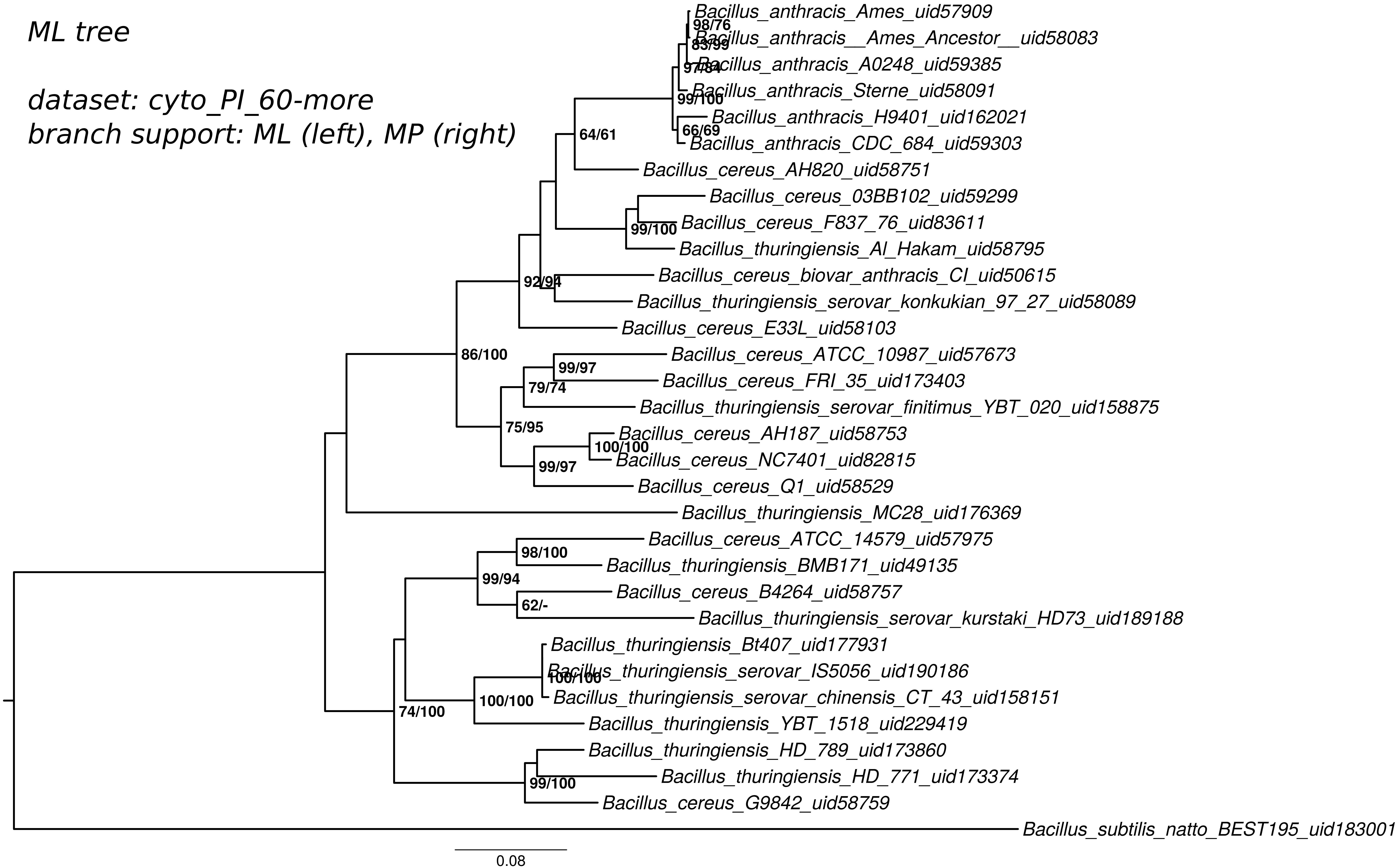
ML tree
dataset: cyto_PI_60-more
branch support: ML (left), MP (right)
Bacillus_anthracis_Ames_uid57909
98/76
Bacillus_anthracis__Ames_Ancestor__uid58083
83/99
Bacillus_anthracis_A0248_uid59385
97/84
Bacillus_anthracis_Sterne_uid58091
99/100
Bacillus_anthracis_H9401_uid162021
66/69
Bacillus_anthracis_CDC_684_uid59303
64/61
Bacillus_cereus_AH820_uid58751
Bacillus_cereus_03BB102_uid59299
Bacillus_cereus_F837_76_uid83611
99/100
Bacillus_thuringiensis_Al_Hakam_uid58795
Bacillus_cereus_biovar_anthracis_CI_uid50615
92/94
Bacillus_thuringiensis_serovar_konkukian_97_27_uid58089
Bacillus_cereus_E33L_uid58103
86/100
Bacillus_cereus_ATCC_10987_uid57673
99/97
Bacillus_cereus_FRI_35_uid173403
79/74
Bacillus_thuringiensis_serovar_finitimus_YBT_020_uid158875
75/95
Bacillus_cereus_AH187_uid58753
100/100
Bacillus_cereus_NC7401_uid82815
99/97
Bacillus_cereus_Q1_uid58529
Bacillus_thuringiensis_MC28_uid176369
Bacillus_cereus_ATCC_14579_uid57975
98/100
Bacillus_thuringiensis_BMB171_uid49135
99/94
Bacillus_cereus_B4264_uid58757
62/-
Bacillus_thuringiensis_serovar_kurstaki_HD73_uid189188
Bacillus_thuringiensis_Bt407_uid177931
Bacillus_thuringiensis_serovar_IS5056_uid190186
100/100
Bacillus_thuringiensis_serovar_chinensis_CT_43_uid158151
100/100
74/100
Bacillus_thuringiensis_YBT_1518_uid229419
Bacillus_thuringiensis_HD_789_uid173860
Bacillus_thuringiensis_HD_771_uid173374
99/100
Bacillus_cereus_G9842_uid58759
Bacillus_subtilis_natto_BEST195_uid183001
0.08

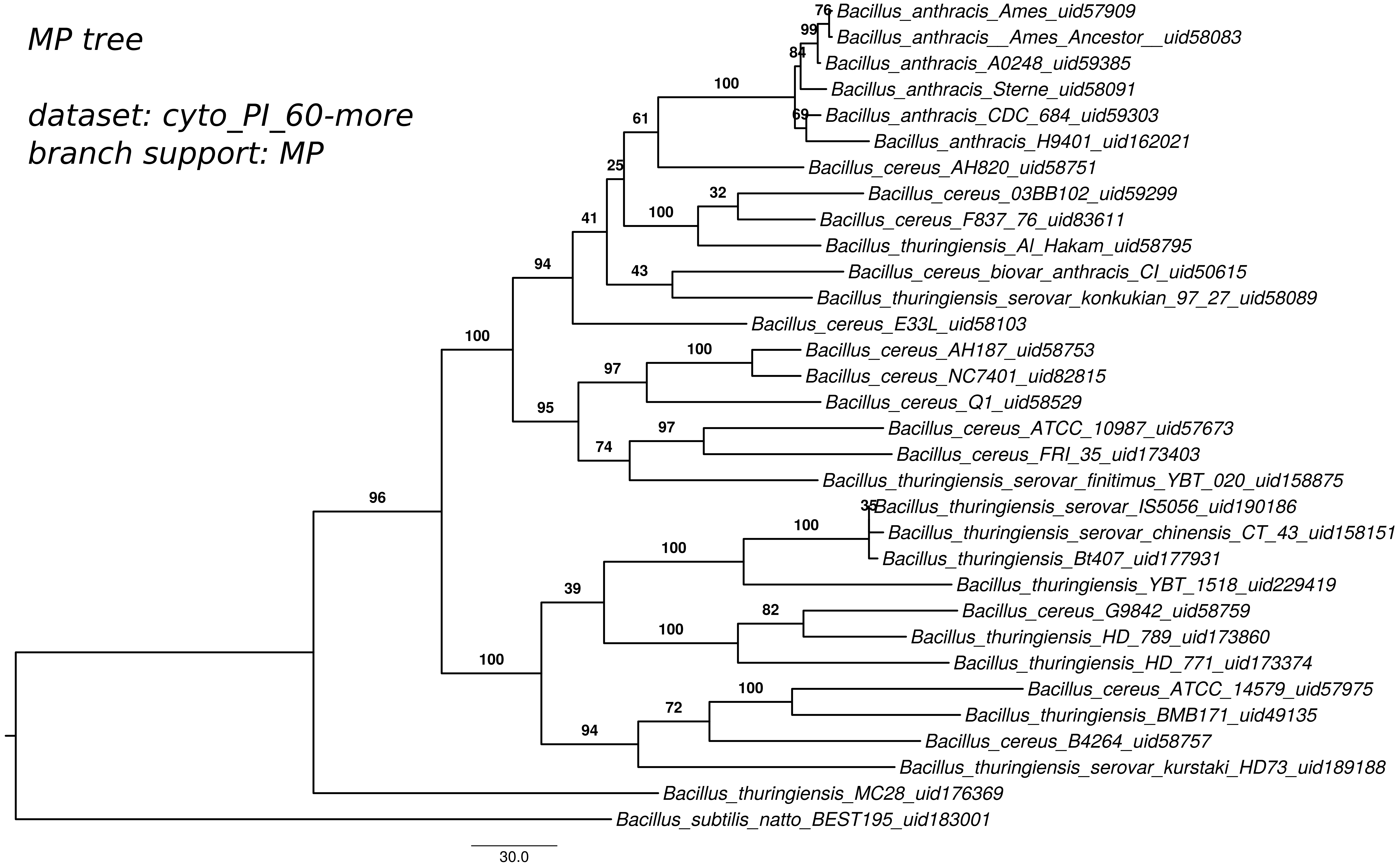
MP tree
dataset: cyto_PI_60-more
branch support: MP
Bacillus_anthracis_Ames_uid57909
Bacillus_anthracis__Ames_Ancestor__uid58083
Bacillus_anthracis_A0248_uid59385
Bacillus_anthracis_Sterne_uid58091
Bacillus_anthracis_CDC_684_uid59303
Bacillus_anthracis_H9401_uid162021
Bacillus_cereus_AH820_uid58751
Bacillus_cereus_03BB102_uid59299
Bacillus_cereus_F837_76_uid83611
Bacillus_thuringiensis_Al_Hakam_uid58795
Bacillus_cereus_biovar_anthracis_CI_uid50615
Bacillus_thuringiensis_serovar_konkukian_97_27_uid58089
Bacillus_cereus_E33L_uid58103
Bacillus_cereus_AH187_uid58753
Bacillus_cereus_NC7401_uid82815
Bacillus_cereus_Q1_uid58529
Bacillus_cereus_ATCC_10987_uid57673
Bacillus_cereus_FRI_35_uid173403
Bacillus_thuringiensis_serovar_finitimus_YBT_020_uid158875
Bacillus_thuringiensis_serovar_IS5056_uid190186
Bacillus_thuringiensis_serovar_chinensis_CT_43_uid158151
Bacillus_thuringiensis_Bt407_uid177931
Bacillus_thuringiensis_YBT_1518_uid229419
Bacillus_cereus_G9842_uid58759
Bacillus_thuringiensis_HD_789_uid173860
Bacillus_thuringiensis_HD_771_uid173374
Bacillus_cereus_ATCC_14579_uid57975
Bacillus_thuringiensis_BMB171_uid49135
Bacillus_cereus_B4264_uid58757
Bacillus_thuringiensis_serovar_kurstaki_HD73_uid189188
Bacillus_thuringiensis_MC28_uid176369
Bacillus_subtilis_natto_BEST195_uid183001
30.0

ML tree

dataset: extracellular
branch support: ML (left), MP (right)

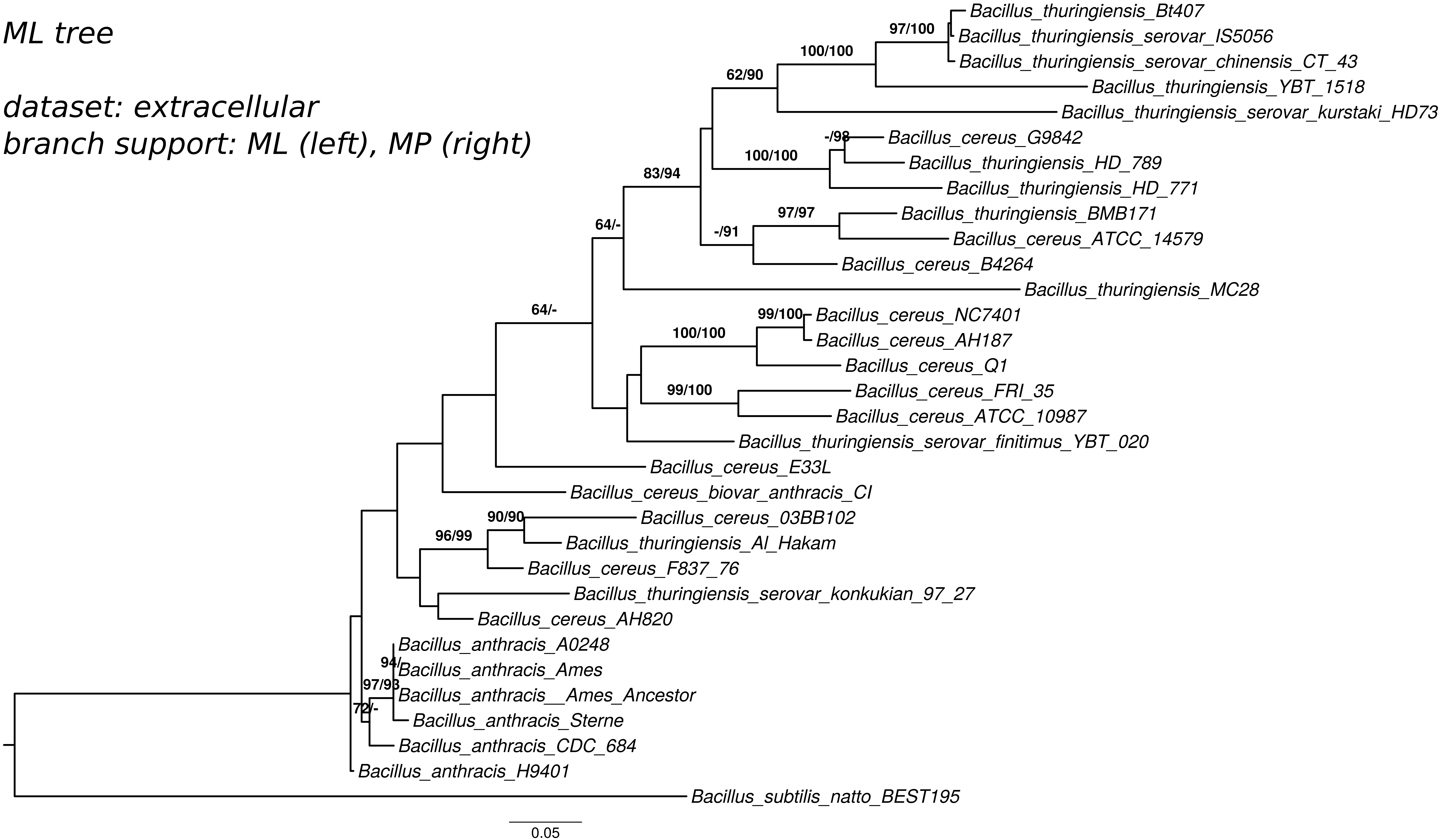

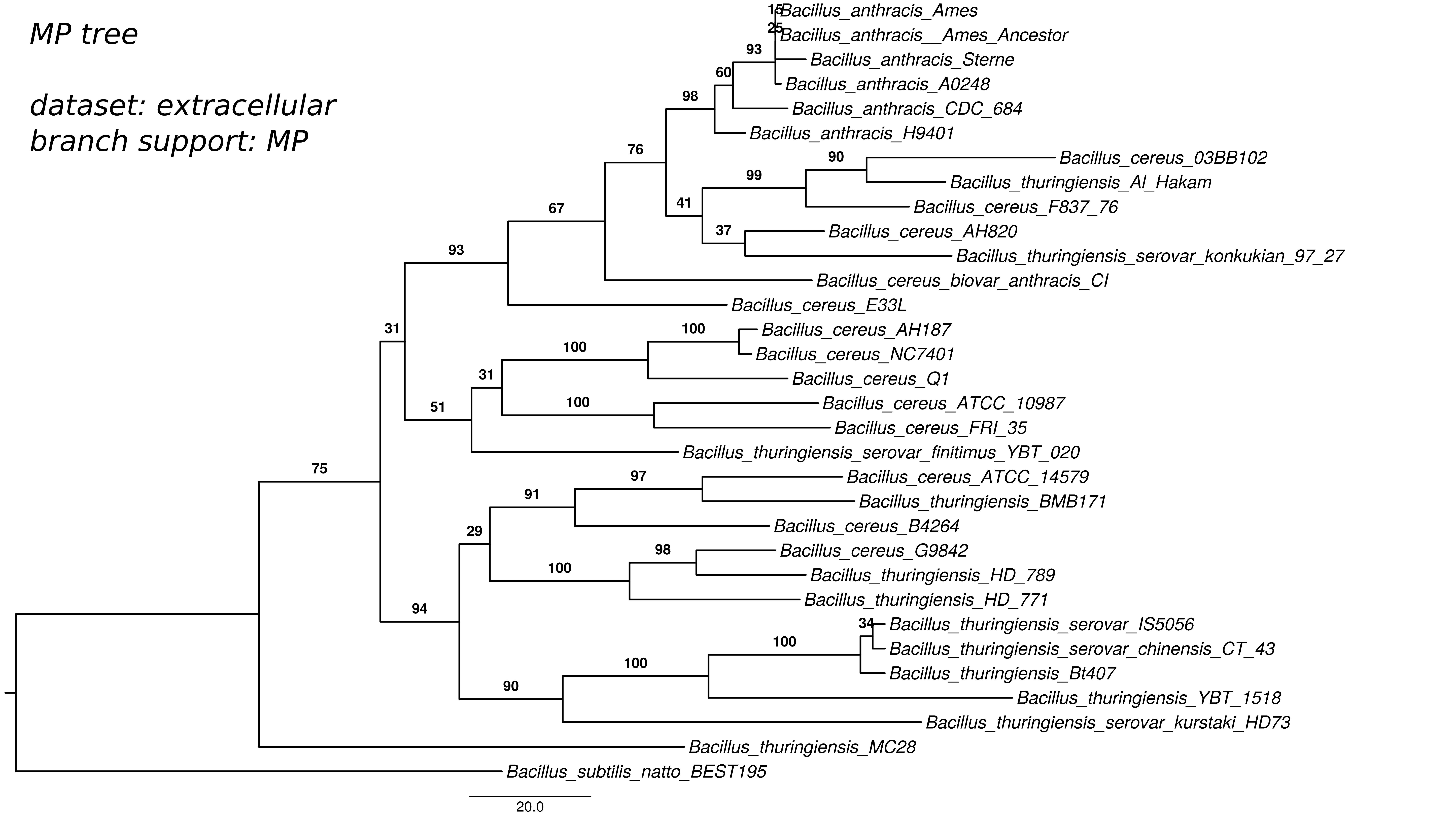
MP tree
dataset: extracellular
branch support: MP
Bacillus_anthracis_Ames
Bacillus_anthracis__Ames_Ancestor
Bacillus_anthracis_Sterne
Bacillus_anthracis_A0248
Bacillus_anthracis_CDC_684
Bacillus_anthracis_H9401
Bacillus_cereus_03BB102
Bacillus_thuringiensis_Al_Hakam
Bacillus_cereus_F837_76
Bacillus_cereus_AH820
Bacillus_thuringiensis_serovar_konkukian_97_27
Bacillus_cereus_biovar_anthracis_CI
Bacillus_cereus_E33L
Bacillus_cereus_AH187
Bacillus_cereus_NC7401
Bacillus_cereus_Q1
Bacillus_cereus_ATCC_10987
Bacillus_cereus_FRI_35
Bacillus_thuringiensis_serovar_finitimus_YBT_020
Bacillus_cereus_ATCC_14579
Bacillus_thuringiensis_BMB171
Bacillus_cereus_B4264
Bacillus_cereus_G9842
Bacillus_thuringiensis_HD_789
Bacillus_thuringiensis_HD_771
Bacillus_thuringiensis_serovar_IS5056
Bacillus_thuringiensis_serovar_chinensis_CT_43
Bacillus_thuringiensis_Bt407
Bacillus_thuringiensis_YBT_1518
Bacillus_thuringiensis_serovar_kurstaki_HD73
Bacillus_thuringiensis_MC28
Bacillus_subtilis_natto_BEST195
20.0
