# Supplementary material for: Improving Phylogeny Reconstruction at the Strain Level Using Peptidome Datasets
Source: PLoS Comput Biol. 2016 Dec 29;12(12):e1005271. doi: 10.1371/journal.pcbi.1005271 (PMC5198984; doi:10.1371/journal.pcbi.1005271)
Supplement: S2 File — Ranges with few instances (e.g., loose ranges of extracellular peptides) and with high abundance (e.g., cytoplasmic proteins consisting of 11 to 20 amino acids) were not helpful for comparison. The analysis focused on extracellular peptides and cytoplasmic peptides consisting of 51 to 60 amino acids and above. (PDF) [file pcbi.1005271.s002.pdf]

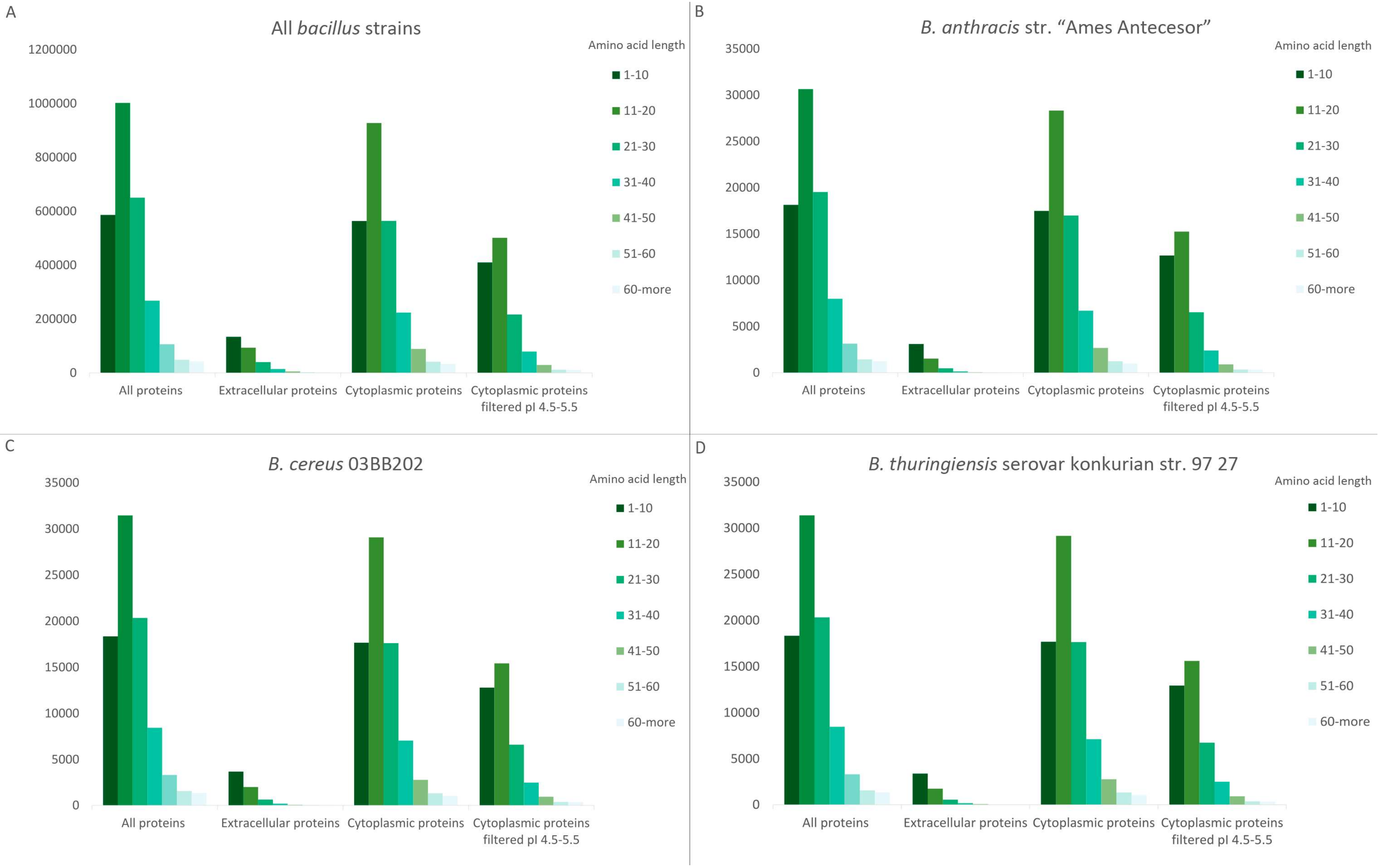
A
All *bacillus* strains
Amino acid length
1-10
11-20
21-30
31-40
41-50
51-60
60-more
1200000
1000000
800000
600000
400000
200000
0
All proteins
Extracellular proteins
Cytoplasmic proteins
Cytoplasmic proteins filtered pI 4.5-5.5
B
B. anthracis str. “Ames Antecesor”
35000
30000
25000
20000
15000
10000
5000
0
C
B. cereus 03BB202
D
B. thuringiensis serovar konkurian str. 97 27
